# Denoising Autoencoders and LSTM-Based Artificial Neural Networks Data Processing for Its Application to Internal Model Control in Industrial Environments—The Wastewater Treatment Plant Control Case

**DOI:** 10.3390/s20133743

**Published:** 2020-07-04

**Authors:** Ivan Pisa, Antoni Morell, Jose Lopez Vicario, Ramon Vilanova

**Affiliations:** 1 Wireless Information Networking (WIN) group, Escola d’Enginyeria, Universitat Autònoma de Barcelona, 08193 Bellaterra, Spain; antoni.morell@uab.cat (A.M.); jose.vicario@uab.cat (J.L.V.); 2 Advanced Systems for Automation and Control (ASAC) group, Escola d’Enginyeria, Universitat Autònoma de Barcelona, 08193 Bellaterra, Spain; ramon.vilanova@uab.cat

**Keywords:** artificial neural networks, denoising autoencoders, long short-term memory cells, internal model control, wastewater treatment plants

## Abstract

The evolution of industry towards the Industry 4.0 paradigm has become a reality where different data-driven methods are adopted to support industrial processes. One of them corresponds to Artificial Neural Networks (ANNs), which are able to model highly complex and non-linear processes. This motivates their adoption as part of new data-driven based control strategies. The ANN-based Internal Model Controller (ANN-based IMC) is an example which takes advantage of the ANNs characteristics by modelling the direct and inverse relationships of the process under control with them. This approach has been implemented in Wastewater Treatment Plants (WWTP), where results show a significant improvement on control performance metrics with respect to (w.r.t.) the WWTP default control strategy. However, this structure is very sensible to non-desired effects in the measurements—when a real scenario showing noise-corrupted data is considered, the control performance drops. To solve this, a new ANN-based IMC approach is designed with a two-fold objective, improve the control performance and denoise the noise-corrupted measurements to reduce the performance degradation. Results show that the proposed structure improves the control metrics, (the Integrated Absolute Error (IAE) and the Integrated Squared Error (ISE)), around a 21.25% and a 54.64%, respectively.

## 1. Introduction

Nowadays, the Digital Transformation is changing the way of understanding the different processes performed in the industrial sector. The Industry 4.0, the Internet of Things (IoT) and the Cyber-Physical Systems have arisen as the main paradigms of this digital transformation. They drive the adoption of new technologies such as data-driven based ones to support and improve the industrial processes. For instance, maintenance or control tasks can be performed automatically relying only on data provided by such a kind of data-driven systems [1].

In that sense, one of the main tools which have arisen as one of the core concepts of the Industry 4.0 paradigm are the Artificial Neural Networks (ANNs) [2]. They are computational models characterised by their capabilities in the modelling of highly complex and non-linear processes performed in several industries such as the chemical one [3]. Besides, one of the most important points of ANNs is that they only need pairs of input and output measurements to model a process. This opens a wide range of processes and activities which can take advantage of these tools. For instance, predictive maintenance or detection of rare events, which are tasks giving highly-valuable information to the companies, can be performed adopting ANNs. One clear example is shown in Reference [4], where ANNs are adopted in the petrochemical industries to automatically detect outliers such as drifts or process disturbances.

Other examples of ANNs applications are the soft-sensors, which are adopted to predict unmeasured variables by means of available data. Besides, the adoption of such sensors entail a reduction in the costs related to sensing activities [5,6,7,8,9]. In References [5,6], ANNs have been considered to implement soft-sensors based on Multilayer Perceptron (MLP) structures. In both cases, the soft-sensors are placed in a refinery debutanizer column to predict the different concentrations involved in the distillation processes. In Reference [5], the MLP has been adopted to implement a Non-linear Autoregressive model while in Reference [6] the nets have been conceived to implement an adaptive linear based soft-sensor to predict multiple components of the distillation column. Another type of industrial processes where soft-sensors have been widely considered corresponds to the sewer systems. In Reference [7], Long Short-Term Memory cells (LSTMs), a topology of Recurrent Neural Networks (RNNs), are considered to model the behaviour of the sewer systems and then manage them. The main idea is to forecast the amount of residual water incoming into the system and then spatially distribute it among the free space of the system.

Another field where the adoption of ANNs is continuously increasing corresponds to the wastewater treatment plants (WWTPs). They consist in industries devoted to reducing the pollutants of the residual urban waters coming from the sewer systems. To perform this, they consider highly complex and non-linear biological and biochemical processes that require some WWTP concentrations at a certain value (set-point). Besides, some control structures are applied in order to manage them. For instance, Proportional Integral (PI) controllers have been considered as the WWTP default control strategy to maintain the dissolved oxygen ($S_{O,5}$) at the fifth reactor tank and the nitrate-nitrogen ($S_{NO,2}$) at the second one [10]. On other cases, Model Predictive Controllers (MPC) and Internal Model Controllers (IMC) have been considered as more complex control strategies improving the default control ones. In Reference [11] MPCs have been adopted to maintain the ammonium effluent concentration at the given values. IMCs are considered in Reference [12], where this structure is proposed to implement two event-based IMC structures devoted to controlling the $S_{NO,2}$ and the $S_{O,5}$. The main point is that these control structures work with events instead of continuous time. Despite their good behaviour and control performance, these control strategies are based on mathematical models of the process under control and therefore, they rely on highly complex and non-linear processes. In that sense, ANNs have been considered in different works to avoid this dependence.

In some cases, ANNs are adopted to predict some measurements to feed the control strategy or to determine certain concentrations required by the control strategies. For instance, in Reference [13], an ANN-based soft-sensor has been proposed to predict three components of the WWTP, the Chemical Oxygen Demand (COD), the Total Nitrogen (TN) and the Total Suspended Solids (TSS). In Reference [14] a soft-sensing model with an adaptive weighted fusion is proposed to compute the quality of the water inside a WWTP. This structure is complemented with wavelet neural networks to model the non-linear WWTP process. In Reference [15], a RNN is considered as a stage performing the predictions of the input and output data required to implement a MPC. Finally, in Reference [16] a MLP net is proposed to model the ammonium ($S_{NH,e}$) and total nitrogen (TN) of a controlled WWTP plant. The idea is to predict those moments where the WWTP is spilling effluent concentrations higher than the ones established by the local administrations. Once an exceed is detected, the control strategy devoted to reducing it actuates accordingly. Finally, in Reference [8], LSTMs have been considered to model the complete behaviour of a WWTP and determine the effluent concentrations. Then a control strategy is applied accordingly to these values.

In terms of the control, ANNs have been considered to implement some control strategies and simplify them as well as their implementation. Some control structures are based on the use of reinforcement learning and adaptive fuzzy neural network controllers [17,18]. For instance, in Reference [18], an adaptive fuzzy neural network has been considered to track the optimal set-points of the dissolved oxygen in the fifth reactor tank and the nitrate-nitrogen of the second one. Other works consider the adoption of the IMC structure due to its easy implementation and good performance [19]. In addition, this structure is very suitable to be used as a decentralised controller since the models required by the IMC can be implemented considering ANNs over cloud-based systems while the process under control is implemented in the real plant. One example of an ANN-based IMC is shown in Reference [20]. There, the controller is based on MLP nets in charge of tracking a variable set-point. Results show that this structure performs well in this task. In addition, it is shown that control structures based on ANNs alleviate the tedious process of designing a control structure since ANN-based models rely on data instead of highly complex mathematical models. Besides, the decoupling of the control actuation from the real processes is also achieved [21,22].

The common point of all these works is that they consider ideal scenarios, that is, situations where WWTP measurements are not corrupted by noise or other non-desired sensor behaviours. Nevertheless, When a real scenario is considered, the ANNs performance as well as the control one drops. To solve this, two different approaches will be proposed here. The former corresponds to the adoption of LSTM nets instead of MLPs in the IMC implementation to exploit the time correlation between measurements. The latter corresponds to a new denoising preprocessing stage which will be proposed to make the controller work with ideal and noise measurements, that is, to be able to work under ideal and real conditions. Two different Machine Learning (ML) approaches, the Principal Component Analysis (PCA) [23] and a Denoising Autoencoder (DAE) [24], will be consider as the denoising stage to clean the incoming measurements. In both cases, they correspond to data-driven systems which simplify the denoising stage implementation with respect to (w.r.t.) conventional solutions which involve the design of filters such as the ones considered in telecommunications receivers or in the satellite image denoising process [25]. The adoption of these data-driven denoising techniques will improve the control metrics in the case where measurements are corrupted by noise. These measurements will be cleaned and therefore, the nets considered in the IMC implementation will be leading with ideal measurements instead of noise-corrupted ones. Furthermore, the stability analysis of the complete ANN-based IMC controller will be performed to determine the range where the IMC structure shows a robust stability.

As a summary, the contributions of this work are:

- Data-driven methods have been considered to model highly complex and non-linear processes.
- A new LSTM-based IMC control strategy has been designed to control the DO of a WWTP plant.
- The stability analysis of the proposed structure is performed to determine if it is suitable to control the DO.
- Three different ML denoising techniques will be considered to clean the noise-corrupted data entering in the LSTM-based IMC structure as well as to simplify the denoising stage implementation.
- The adoption of the whole structure, (LSTM-based IMC and the denoising stage) helps in the improvement the control metrics: the Integrated Absolute Error (IAE) and the Integrated Squared Error (ISE) are improved a 21.25% and a 54.64%.

Finally, the rest of the paper is structured as follows—in Section 2 the material and methods considered in this work are presented—the Benchmark Simulation Model No.1 (BSM1), the ANN-based IMC structure and the PCA, one of the denoising methods, are defined in this section. In Section 3 the LSTM nets considered in the ANN-based IMC implementation and the Denoising Autoencoders considered here are described. Then, results of the different tests performed to the proposed IMC structures are shown in Section 4. They consist in the prediction tests to determine the prediction accuracy of the considered ANNs, the stability test to determine the range of robust stability and the control test to determine the behaviour of the whole IMC structure. Finally, Section 5 concludes the paper.

## 2. Material and Methods

### 2.1. BSM1 Framework

In this work we are proposing the implementation of an ANN-based IMC controller able to work in ideal and real scenarios. For that purpose, a new denoising preprocessing stage based on ML techniques will be proposed. The case under study, where the new approaches will be deployed, consists in a WWTP plant. Since there exists a great range of WWTP topologies and architectures, this work is based on the Benchmark Simulation Model No.1 (BSM1) [26].

BSM1 model simulates the behaviour of the water line of a general purpose WWTP devoted to reducing the nitrogen derived pollutant components of residual urban waters [26]. To perform this, BSM1 follows the mathematical models described in the Activated Sludge Model No. 1 (ASM1) which is defined by the International Water Association (IWA) [27]. Besides, WWTP plants also are able to reduce other pollutant components such as phosphorus derived ones. The processes in charge of reducing these pollutants are defined in the Activated Sludge Models No. 2, No. 2d and No. 3 (ASM2, ASM2d and ASM3, respectively) [28], however, they are not implemented in the BSM1 model [26].

In such a context, BSM1 framework can be understood as a Digital Twin of a WWTP plant, that is, a digital model which describes the functionality and behaviours of a real system following mathematical models [29]. Although BSM1 has been designed to simulate a general purpose WWTP, it can be modified accordingly to a real plant in order to simulate its behaviour. Therefore, any change or test can be performed in the digital twin without compromising the behaviour of the real plant. This makes sense in the design process of any new control strategy. Instead of modifying the architecture of the real plant, one can change, modify and test the new control approach in BSM1. Therefore, the interruption of the processes carried out in the real plants and their consequences can be avoided [30].

#### 2.1.1. BSM1 Architecture

**Figure 1:**
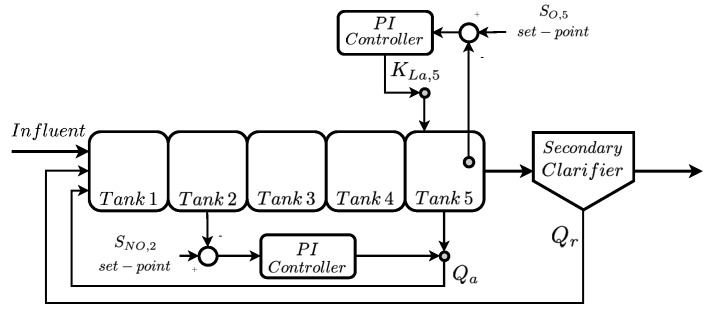
Benchmark Simulation Model N.1 (BSM1) model for a biological wastewater treatment plant (WWTP). $Q_{a}$ and $Q_{r}$ are the internal and external recirculation flow rates.

BSM1 architecture is designed to process a total influent volume of 18,446 m^3^/day. It will be treated in the reactor tanks, which are in charge of performing the different biochemical and biological processes described in the ASM1 models [26,27]. In this case, five reactor tanks are considered: two anoxic working with a lack of oxygen and three aerated. Just after the reactor tanks, the BSM1 model implements a secondary clarifier before spilling the treated water to its natural cycle (see Figure 1). Finally, the whole BSM1 structure has a volume of 12,000 m^3^ distributed as follows: (i) 1000 m^3^ each anoxic tank, 1333 m^3^ each aerated tank and 6000 m^3^ the secondary clarifier. These volumes defines a water retention time around 14.4 h, that is, incoming influent will last around 14 h to get out of the plant [26]. In that sense, BSM1 model considers a total amount of 15 concentrations which are involved in the pollutant reduction process. More details of the 15 variables considered by the BSM1 model can be observed in Reference [10]. They are modified at each reactor tank accordingly to the processes defined in Reference [27] and their evolution can be observed after each stage of the BSM1 model, that is, after any reactor tank or clarifier.

In terms of the reactor tanks behaviour, the anoxic tanks are in charge of the denitrification processes which transform the nitrate components into nitrogen gas ($N_{2}$) and its derivates ($NO_{2}$). The aerobic tanks transform the ammonia ($S_{NH}$) into nitrates by means of the nitrification process [31]. In order to perform both processes in a correct manner, some concentrations of certain components have to be maintained at certain levels. This is the case of the dissolved oxygen ($S_{O,5}$) in the fifth reactor tank and the nitrate-nitrogen in the second tank ($S_{NO,2}$) [10]. In this case, BSM1 implements two simple control strategies based on Proportional Integral (PI) controllers [32]. The former is devoted to maintain the $S_{O,5}$ at a constant value (set-point) of 2 mg/L while the latter is in charge of assuring a $S_{NO,2}$ level equal to 1 mg/L. The PI controlling the $S_{O,5}$ will modify the oxygen transfer coefficient of the fifth reactor tank ($K_{La,5}$) in order to maintain the set-point at the given value. The PI controlling the $S_{NO,2}$ will modify the internal recirculation ($Q_{a}$) flow rate accordingly to the observed nitrate-nitrogen.

#### 2.1.2. BSM1 Simulation Protocol

BSM1 not only works as a Digital Twin of a general purpose WWTP, but also as a benchmark where different control strategies can be tested. To do so, BSM1 defines a simulation protocol to assure that control strategies will be compared under the same circumstances. This protocol consists in the simulation of a 14 day influent profile sampled every 15 min, that is, adopting a sampling frequency ($f_{s}$) equal to 1.11 mHz. BSM1 provides three influent profiles as a function of the weather it represents [33,34]:

- Dry Weather: 14 days of dry weather considering daily variations.
- Rainy Weather: Dry weather adding a long rainy episode during days 9 and 10.
- Stormy Weather: Dry weather adding two storm episodes at days 8 and 11.

Before applying these influent profiles, the BSM1 model has to be initialized. This is performed simulating a 100-days constant influent profile. The objective of this simulation is to carry the BSM1 model to its steady-state, that is, the point where the system is stabilised. Once the benchmark reaches this point, the different control strategies can be compared against other strategies in terms of the control itself. For that reason two metrics are defined, the Integrated Absolute Error (IAE) and the Integrated Square Error (ISE). These metrics are only computed considering the last seven days of the simulation, that is, those samples showing the behaviour of the WWTP from day 7 to day 14:

(1) $$IAE=\sum_{n=7\mathrm{th}\;\mathrm{day}}^{10\mathrm{th}\;\mathrm{day}}\left|e\left[n\right]\right|\;ISE=\sum_{n=7\mathrm{th}\;\mathrm{day}}^{10\mathrm{th}\;\mathrm{day}}{\left(e\left[n\right]\right)}^{2},$$

where e[n] is the difference between the given set-point and the measured concentration.

In terms of the measurements, it is worth noting that BSM1 implements sensors for different chemical products such as the ones considered in the BSM1 default control strategies. Each sensor also includes a noise option just to determine if it will add noise to the measurements or not. This noise is defined as a white zero mean gaussian noise with unity variance multiplied by the defined noise level, that is, 2.5% of the maximum value sensed by the corresponding sensor [10]. More specific noise has not been considered in BSM1 model since this will entail that different types of sensors would not be comparable among them [10]. All of this motivates two types of tests—(i) and ideal test where noise is not considered and (ii) a realistic one where all the sensors implemented in the BSM1 model are adding noise.

### 2.2. ANN-Based Internal Model Control Structure

**Figure 2:**
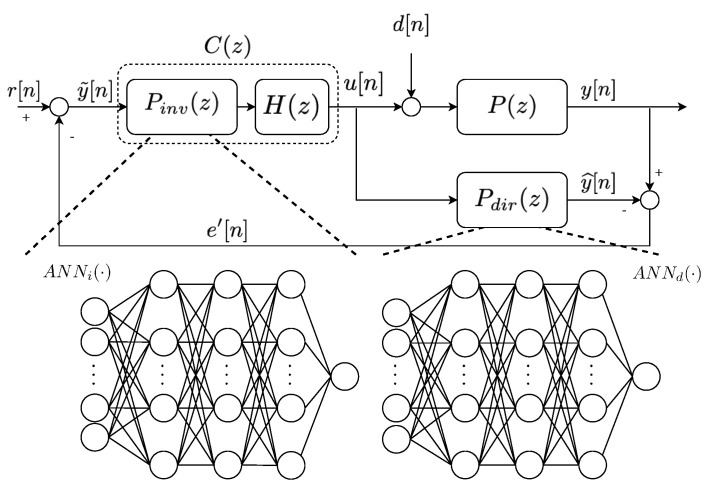
Internal Model Controller (IMC). r[n] corresponds to the reference signal, $e^{\prime }\left[n\right]$ to the mismatch between the output of the real process and $P_{dir}\left(z\right)$ output ($\hat{y}\left[n\right]$).

As has been previously stated, this work is based on the application of an IMC structure based on ANNs to substitute the default PI controlling the $S_{O,5}$ concentration. The adoption of ANN-based IMC structures is motivated due to their ability to deal with non-linear processes as well as to their good performance when dealing with tracking process [12,19,20]. This is possible thanks to the ANNs considered in the IMC implementation. In this case the direct ($P_{dir}\left(z\right)$) and the inverse ($P_{inv}\left(z\right)$) models of the IMC structure are implemented by ANNs (see Figure 2). Therefore, if $ANN_{d}\left(\cdot \right)$ and $ANN_{i}\left(\cdot \right)$ are defined as the ANN modelling $P_{dir}\left(z\right)$ and $P_{inv}\left(z\right)$, the actuation (u[n]) and the controlled (y[n]) signals will be forecasted as follows:

(2) $$\hat{y}\left[n\right]=ANN_{d}\left(u\left[n\right]\right)\;u\left[n\right]=H\left(ANN_{i}\left(e^{\prime }\left[n\right]\right)\right),$$

where H(z) is a first-order filter which reduces the effects of non-desired behaviours such as non-linearities or non-invertible parts. Its cut-off frequency will be computed by means of the stability test performed to the whole control strategy.

The main point of the ANN-based IMCs is that they do not require a mathematical model of the process under control any more. Consequently, they become purely data-driven approaches. In addition, the decoupling of the control strategy from highly-complex and non-linear systems is achieved. Some examples where ANN-based IMCs are considered corresponds to References [20,21,22]. In Reference [21] the ANN-based IMC is implemented to decouple the control of an autonomous vehicle from its model while in Reference [22], the IMC is considered to regulate the light of some workstations of an office. Finally, in Reference [20], an ANN-based IMC implemented with MLPs has been deployed over a WWTP to control the dissolved oxygen of the last reactor tank.

However, one of the biggest drawbacks of the ANN-based IMCs is that they completely rely on the pairs of input/output data considered in their training process: the more precise the measurements, the better the control performance. Consequently, it is important to gather measurements of the real process with the minimum possible variations. If an ideal scenario is considered, the measurement will be directly the most accurate ones. On the other hand, in real scenarios measurements are corrupted with noise added by non-ideal sensors. This entail that ANNs trained with ideal measurements are no longer valid because of the noise effect. Thus, this effect has to be mitigated if ANNs are going to be adopted in the IMC control design. This can be performed either training the ANNs with noisy signals or adopting a denoising stage. The former approach implies that we have to design different ANNs for each scenario whereas the latter implies that the ANNs considered in the ideal scenario are valid for the real one.

For that reason, in the case under study (a WWTP plant) two different scenarios have been defined to design the control approaches and to determine their behaviour:

- Ideal Scenario: The default PI controlling the $S_{O,5}$ concentration will be substituted by an IMC structure based on ANNs. Specially, two types of ANNs have been considered, the MLPs and the Long-Short Term Memory (LSTM) cells. In both cases, they model the direct and inverse relationships of the process under control. The difference between this scenario and the others is placed in the sensors it considers: they are ideal and therefore, they sense the different involved concentrations neither adding noise nor delays.
- Real Scenario: This scenario considers the same IMC control strategy as in the ideal case. However, the sensors implemented here add noise and delays to the measurements. Since the proposed IMC control strategy is implemented with ANNs, it highly depends on the measured data. One consequence is directly observed in the controller performance which drops when noise corrupted measurements are considered. For that reason, a new denoising preprocessing stage has been implemented here. This denoising stage will be based on data-driven methods such as ML techniques or ANNs. The ones considered here are the Principal Component Analysis (PCA) [23] and the Denoising Autoencoders (DAE) [24]. Moreover, the proposed denoising stage can be understood as as a complement of the ANN-based IMC structure controlling the BSM1 $S_{O,5}$ component. It it not only adopted in this case, but also it can be extensible to different situations where a cleaning of noisy data is required.

Here, the IMC structure will be based on the control strategy presented in Reference [20]. Nevertheless, the ANN-based IMC will consider LSTM nets instead of MLPs and will be also deployed over the two previously stated scenarios, that is, the ideal and the real ones. Moreover, the stability analysis of the different control approaches will be computed to determine if the control strategies can be applied or not.

### 2.3. LSTM Cells

In the case under study, the process under control corresponds to the biological and biochemical processes performed in the fifth reactor tank of the WWTP plant. Since these processes are highly complex and non-linear, the adoption of ANNs is highly encouraged ([35], Chapter 6). Among the different network topologies, the models can be implemented by means of MLP networks ([36], Section 2.2) and Long Short-Term Memory (LSTM) cells ([35], Section 10.10). The control approach considering MLP nets have been previously implemented and analysed in Reference [20]. Results have shown that these nets are offering a good prediction and control performance. However, the stability analysis of the control strategy was not carried out.

In this work, the IMC structure will be implemented considering LSTM cells instead of MLP ones. These nets are characterised by their suitability when dealing with time-series signals such as voice or text ([35], Chapter 10). Here, signals corresponds to different concentration measurements which are sensed every 15 min. Therefore, they form a time-series signal which not only depends on the input concentrations entering in the plant at this moment, but also in the past values. Thus, they are highly-correlated in time which motivates even more the adoption of LSTM cells to model $P_{inv}\left(z\right)$ and $P_{dir}\left(z\right)$ [37].

**Figure 3:**
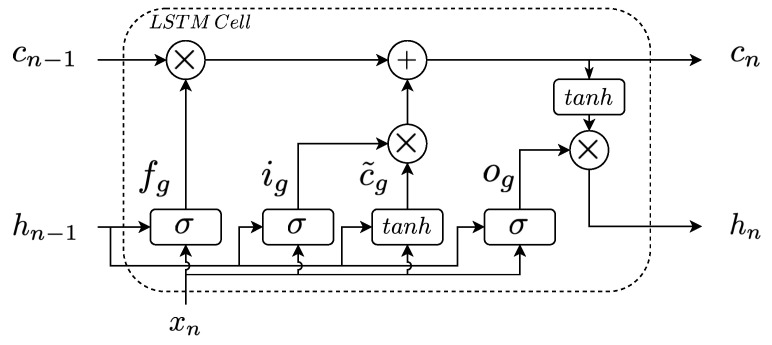
Long short term memory (LSTM) cell structure.

The ability of LSTM to deal with time-series comes from the fact that they implement a memory cell which stores information about the previously observed measurements. To manage the memory, they also implement four different gates (sigmoid and hyperbolic tangent layers) More details about the behaviour and implementation of the LSTM cell can be observed in ([35], Section 10.10). The gates corresponds to the input, the forget, the cell state-update and the output gates (see Figure 3):

- Forget gate ($f_{g}$): the forget gate determines which information of the cell state ($c_{n}$) has to be taken into account in the new state computation. To determine this, it considers the actual input information ($x_{n}$) and the output of the previous time-step ($h_{n-1}$). This gate considers a sigmoid layer ([36], Chapter 1) which yields values between 0 and 1, where 0 refers to completely forget the cell state and 1 refers to keep it and modify the hidden state accordingly to the gate’s output.
- Input gate ($i_{g}$): the cell state ($c_{n}$) is modified with the output computed by the input gate ($i_{g}$) and the cell-state update (${\tilde{c}}_{g}$). The input gate performs the same behaviour as the forget gate, however, it will determine if the input information has to be considered in the new cell state or not.
- Cell-state gate (${\tilde{c}}_{g}$): the cell-state gate computes the new cell state candidate values considering the previous output and the input of the cell. Here, the activation function corresponds to an hyperbolic tangent which yields values between −1 and 1. Finally, the new cell state ($c_{n}$) is computed as a combination of the three previously stated gates ($f_{g}$,$i_{g}$ and ${\tilde{c}}_{g}$).
- Output gate ($o_{g}$): the output gate determines the cell output candidates. These are modified accordingly to the cell state in order to obtain the output of the cell ($h_{n}$). The output is computed as a function of the input data ($x_{n}$), the previous output ($h_{n-1}$) and the actual cell-state ($c_{n}$).

### 2.4. Stability Analysis

The stability of control strategies has to be computed to determine if it will be stable or not in the range of the working frequencies. In the case of ANN-based IMC structures, they are implemented with ANNs and therefore, they no longer rely on mathematical models. For that reason, the stability of the control strategy can not be determined with conventional approaches which require mathematical expressions or transfer functions of the models involved in the control. For that reason, the stability of the whole IMC structure will be computed adopting the Empirical Transfer Function Estimation (ETFE)-based strategy analysis defined in Reference [38]. It is based on the estimation of the transfer functions of the different elements involved in the IMC structure. Then, the stability test is performed considering that a system is robustly stable if it follows the following criterion:

(3) $$|P_{dir}\left(e^{j\omega }\right)C\left(e^{j\omega }\right)l_{m}\left(\omega \right)|\leq 1,$$

where $P_{dir}\left(e^{j\omega }\right)$ and $C(e^{j\omega })$ are the estimated transfer functions of $P_{dir}\left(z\right)$ and $P_{inv}\left(z\right)\cdot H\left(z\right)$, respectively. $l_{m}\left(\omega \right)$ corresponds to the inverse of the multiplicative uncertainty bound, which is computed as explained in References [38,39]. In Reference [38], the ETFE-based analysis is performed to determine the stability of an event-based IMC structure. In Reference [39], this method is applied to determine the stability of an ANN-based IMC structured implemented with LSTM cells. However, this ANN-IMC is tested in an ideal scenario where measurements are neither corrupted by noise, nor by delays. Here the same principle is considered, however, the ETFE-based stability analysis will not be only applied in an ideal scenario, but also in a real one, where the measurements are corrupted by noise and delays. Results will show that stability drops if no action is performed to alleviate the problems derived from the appearance of noise and delays. This is solved by means of a denoising approach whose implementation and behaviour are described along this work.

### 2.5. PCA Denoising Method

The main aim of PCA is to reduce the dimensionality of the input data mapping them into a latent space with a lower dimension ([35,40], Chapter 14). The same principle is applied when the PCA algorithm is considered as a denoising method. In this case, it deals with noisy signals which can be interpreted as time-series signals showing a high correlation in time, however, they are corrupted by noise [26]. To denoise them, a PCA denoising strategy is followed. The noise-corrupted signals are mapped into the PCA latent space and then they are recovered considering only the eigenvalues of the signal space [23]. Besides, a sliding window (SW) has also been considered to sort the measurements in time and preserve their correlation [23]. However, although PCA can be adopted as a denoising method, its denoise performance can be compromised due to the nature of the system—PCA has been conceived to work with linear systems while WWTPs are non-linear ones [26]. Consequently, more complex denoising approaches such as the Denoising Autoencoders have to be considered [24].

### 2.6. Modelling

The functionality and behaviour of the different data-driven methods (ANNs and denoising processes) have been modelled adopting three different tools: Simulink, Matlab and Python. The BSM1 framework model has been implemented over Simulink [26]. For instance, the input and output data considered in the ANNs training process have been generated performing a whole year simulation in Simulink. It has been also considered to implement the final version of the LSTM-based IMC controller and its denoising process. Matlab has been considered to design the PCA-based denoising approach. Python 2.7 has been adopted to design, implement and train the neural networks considered not only in the ANN-based IMC controller, but also in the implementation of the denoising approaches based on ANNs. The different libraries that have been considered are:

- Scikit-Learn (0.20.1) [41]: Scikit-Learn is a Python library which implements a great number of machine learning algorithm and functions. For instance, the normalisation and denormalisation processes, the division of data into training and test datasets as well as the K-Fold cross-validation technique and the Autoregressive Integrated Moving Average (ARIMA) [42] models have been implemented with this library. Not only this, the metrics considered to compute the prediction performance have been also taken from this library.
- NumPy (1.15.4) [43]: NumPy is one of the well-known Python libraries. It offers solutions to deal with vector and matrices.
- Pandas (0.23.4) [44]: Pandas library corresponds to a Python library which has arisen as an extension of NumPy library. Pandas is adopted to deal with data tables and time-series. Besides, it is adopted to load different types of data into Python.
- Matplotlib (2.2.2) [45]: Matplotlib corresponds to a Python library specialised in performing different types of plots, for instance, the correlation matrix and the training curves.
- Tensorflow (1.12.0) [46]: Tensorflow is an open-source machine learning framework specialised in the implementation of neural networks and deep learning structures and algorithms. In terms of the neural networks, Tensorflow allows their implementation from scratch or by means of the Keras API, which implements the architectures of neural networks from predefined structures. All the Tensorflow instructions have been executed over a NVIDIA${}^{®}$ GeForce RTX 2080 Titan GPU memory.

## 3. Denoising LSTM-Based IMC

BSM1 considers two possible scenarios—(i) the ideal one where the sensors measure the different WWTP concentrations without adding any non-desired behaviour and (ii) the real scenario where the sensors are real and therefore, add noise to the measurements. As a consequence, the ANN-based IMC has to be designed accordingly to the scenario where it is going to be applied. In the case of the ideal scenario results show that two LSTM-based architectures are enough to achieve a good control performance. However, this completely changes when noise is introduced in the scenario (real scenario). To overcome this, two approaches have been considered—(i) the retraining of the LSTM-based architectures considering the measurements of the new scenario and (ii) the implementation of a denoising stage as an extra layer to clean the noise-based measurements and then consider the LSTM-based architectures of the ideal case.

### 3.1. LSTM Architectures

**Figure 4:**
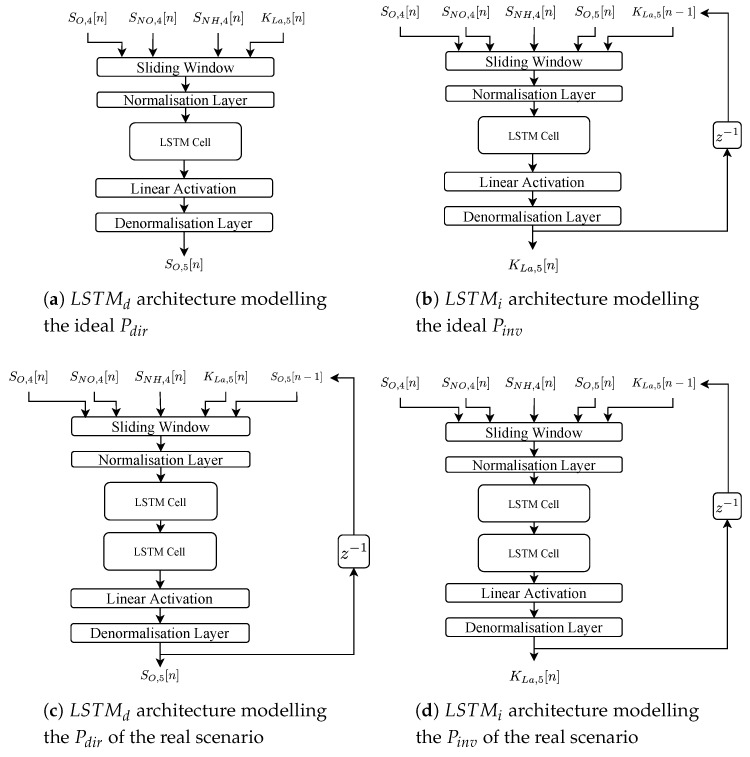
LSTM Architectures considered in the LSTM-based IMC controller. $S_{O,4}\left[n\right]$, $S_{NO,4}\left[n\right]$ and $S_{NH,4}\left[n\right]$ are the oxygen, the nitrate-nitrogen and the ammonium concentrations in the fourth reactor tank.

Four architectures, two per scenario, have been proposed. They follow the same structure: a data pre-processing stage where a sliding window (SW) and a normalisation layer (NL) are implemented, the LSTM cells themselves, a linear activation layer (LAL) and a denormalisation layer (DL) (see Figure 4). The LSTM architectures of the ideal scenario consider a unique LSTM cell whereas the two architectures of the real scenario (LSTMs retrained considering measurements corrupted by noise) consider two cells.

The SW is considered to sort the values in time and therefore, save information about the time-dependence of the input data. It is characterised by two parameters, the window length and the horizon of prediction. The former equals to the lowest periodicity of the LSTM input signals. To determine it, the Fast Fourier Transform is applied instead of an heuristic method. In that manner, the lowest periodicity can be computed regardless of whether input signals are affected by noise or not: the highest input signal frequency component will determine the lowest periodicity and therefore, the minimum SW length to consider. In our case, this periodicity is given by the ammonium concentration in the fourth tank $S_{NH,4}$ and equals to 4 h. For that reason the SW is set to a total amount of 4 h. Besides, this will entail that the LSTMs will be trained considering at least the minimum periodicity of their input signals. Lower window lengths will entail a poor training due to the fact that not enough information from the input signals is considered. On the other hand, if large window lengths are considered instead, there will be enough information to train the networks at expense of increasing their training time. Thus, a trade-off is present—the higher the sliding window, the higher the training time. To solve it, the minimum periodicity of input signals is considered here. The horizon of prediction is set to one sample since the main aim of the LSTM nets is to forecast the actuation variable and the corresponding $S_{O,5}$ value without any type of delay. The NL is in charge of reducing the heterogeneity of input data by means of normalising the input data towards zero mean and unit variance [47]. For instance, the $S_{NO,4}$ input concentration is in the [0,20] mg/L range while the $K_{La,5}$ is in the [0,360] day^−1^ range. The DL transforms the normalised values into their real range. Finally, a LAL has been considered in this work since we are dealing with a regression problem ([36], Chapter 1).

Input data vary depending on the architecture purpose—the LSTMs modelling the direct relationship will consider the actuation signal as an input while the controlled signal corresponds to the output. On the other hand, the LSTMs modelling the inverse relationship forecast the actuation signal considering the controlled one. The actuation and controlled signals are the following ones:

- Actuation Signal (u[n]): the signal entering in the real plant and the IMC’s direct model. It corresponds to the $K_{La,5}$. Physically, it is directly related to the opening of the valve in charge of the aeration of the fifth reactor tank. The larger the $K_{La,5}$, the higher the aeration of the tank [26].
- Controlled Signal (y[n]): it corresponds to the $S_{O,5}$. The IMC structure will be in charge of maintaining the $S_{O,5}$ at the given set-point (r[n]) by means of modifying the $K_{La,5}$.

**Figure 5:**
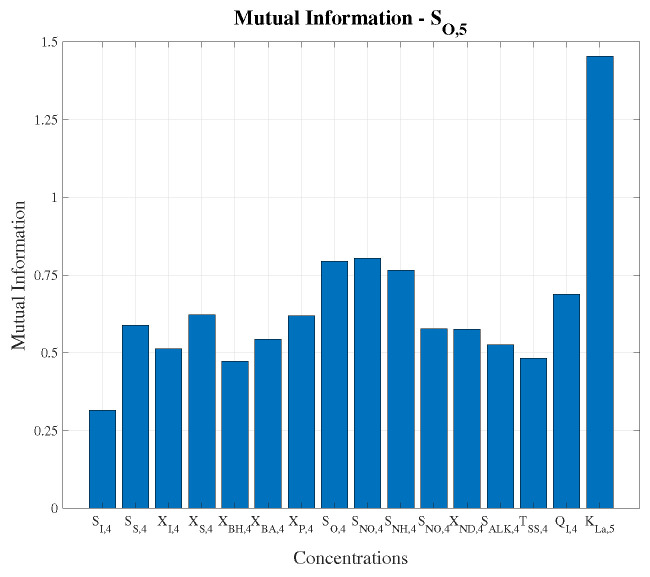
Mutual Information between input data. The other concentrations corresponds to BSM1 influent concentrations: the soluble and particulate inert organic matter concentration ($S_{I}$ and $X_{I}$), the readily and slowly biodegradable substrate concentration ($S_{S}$ and $X_{S}$), the active heterotrophic and autotrophic biomass concentration ($X_{BH}$ and $X_{BA}$), the concentration of particulate products arising from biomass decay ($X_{P}$), the soluble and particulate biodegradable organic nitrogen concentration ($S_{ND}$ and $X_{ND}$), the alkalinity ($S_{ALK}$) and the input flow rate ($Q_{i}$).

Moreover, the LSTM-based architectures have been provided with more information coming from the WWTP plant. This extra information has been selected considering the mutual information (MI) and correlation between the BSM1 concentrations entering in the fifth reactor tank and the controlled variable. Thus, those measurements showing the highest correlation and MI with the controlled signal will be the ones considered to complement the LSTM input data [48,49]. Results in Figure 5 show that among the 15 possible measurements given by the BSM1 model [26], the dissolved oxygen ($S_{O,4}$), the nitrate-nitrogen ($S_{NO,4}$) and the ammonium ($S_{NH,4}$) are the concentrations yielding the highest MI with the $S_{O,5}$ concentration.

#### LSTM Hyperparameters Tuning

The architectures shown in Figure 4 have been found adopting a grid search methodology. In it, different hyperparameters of the LSTM cell such as the number of cells, the amount of hidden neurons per gate (hidden neurons per sigmoid or hyperbolic tangent layer) and the learning rate have been determined. Not only this, two topologies of architectures have been considered as well. The former corresponds to the most simple architecture, which mainly consists of LSTM cells. The latter, however, implement a feed-back connexion of the predicted value. This type of nets are also known as Non-linear Autoregressive Exogenous Model (NARX), which not only takes into account the current and past values of the input signals, but also the previously predicted ones [50]. In such a context, the LSTM architectures obtained after performing the grid search (see Figure 4) are as follows:

- Ideal Direct LSTM net: considers a unique LSTM cell with 100 hidden neurons per gate.
- Ideal Inverse LSTM net: considers a unique LSTM cell with 100 hidden neurons per gate. In this case, the architecture implements the NARX principle, that is, the one-sample delayed $K_{La,5}$ predicted value is considered as one input signal.
- Real Direct LSTM net: considers two stacked LSTM cells with 100 hidden neurons per gate. This architecture follows the NARX structure.
- Real Inverse LSTM net: considered two stacked LSTM cells with 100 hidden neurons per gate. The NARX structure is also considered.

All the architectures adopt a learning rate equal to $1\cdot 10^{-3}$. Once the architectures have been obtained, they have been cross-validated by means of K-Fold (with 5 folds) method to fine-tune the regularisation parameters [51,52]. Regularisation techniques have been applied since LSTM nets are prone to show overfitting. L2 extra-penalty ([35], Sections 7.1 & 7.2) and early-stopping ([35], Section 7.8) methods have been considered to overcome this effect. All the nets have been trained adopting the Back Propagation Through Time (BPTT) algorithm with Adam optimizer ([35], Section 8.5.3). In our case, L2 extra penalty has been set to $1\cdot 10^{-3}$ for the ideal LSTM nets while it equals to $1\cdot 10^{-4}$ for the real ones. In terms of the epochs, a total amount of 500 epochs have been considered. However, they are not always reached in the training process since Early Stopping acts before.

Four datasets have been considered to perform the hyperparameters tuning process: two of them obtained from the ideal scenario and two from the real one, that is, from BSM1 model adding AWGN noise to the different measurements [10]. At each scenario, one dataset is considered to perform the grid-search process while the other is considered to cross-validate the networks. The dataset devoted to cross-validating the architectures is split in two main parts: (i) an 85% of the data is considered to perform the cross-validation process; and (ii) the remaining 15% is leaved for testing purposes. In terms of the measurements, they correspond to the fifth reactor tank input and output concentrations observed during a year. To obtain them, a whole year simulation of different influent profiles has been performed in Simulink. These profiles correspond to uniformly distributed dry, rainy and stormy weather influents. Measurements have been gathered with a sampling frequency of 1.11 mHz, or in other words, four samples per hour. The sensors considered for each variable are defined in the BSM1 model. They are characterised by the delay and rise time as well as by the standard deviation of the AWGN noise they are introducing [10]. Once the different ANN-IMC structures are trained, they are tested over the two defined scenarios, the ideal one (BSM1 scenario) and the real one (BSM1 model + AWGN noise).

### 3.2. Denoising Stage

**Figure 6:**
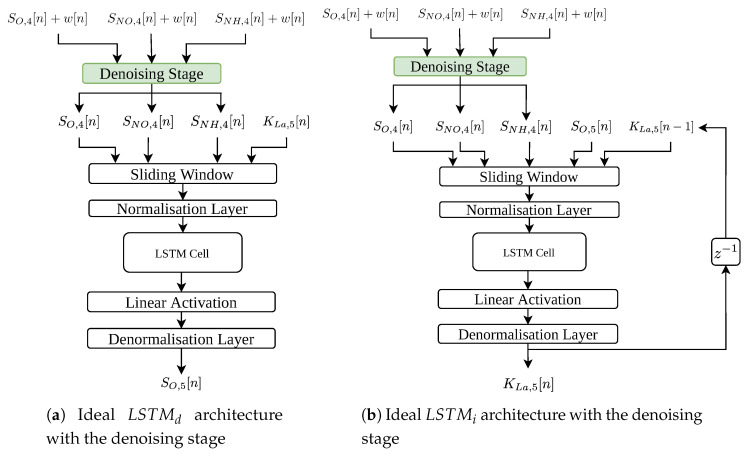
Ideal LSTM architectures with the Denoising Stage.

The denoising stage has been implemented considering two different approaches. The first one corresponds to the PCA (see Section 2.5), which is a well-known ML algorithm adopted to reduce the dimensions of input data in linear problems ([35], Chapter 13). The other method is based on the application of Autoencoders, an ANN-based structure devoted to reducing the input dimensions of data as well ([35], Chapter 14). Among the different Autoencoder topologies, the denoising stage will be based on the Denoising Autoencoder (DAE) network, which receives noise corrupted measurements and transform them into ideal ones ([35], Section 14.5). In that sense, the denoising stage will be considered as a previous layer of the ideal LSTM-based architectures (see Figure 6a,b).

#### 3.2.1. Denoising Autoencoders

Denoising Autoencoders (DAE) are an extension of the well-known Autoencoder structure, which can be observed as a PCA able to deal with non-linear processes ([35], Chapter 14). The Autoencoder structure corresponds to a neural network whose main objective is to replicate the input data in the output layer. This net is characterised by its structure, which has two well-defined parts: an encoder and a decoder. The former is in charge of extracting characteristics of the input data and then map them into a latent space with a reduced dimensionality. The decoder performs the inverse process: it generates data taking into account measurements of the latent space and transform them into the real space taking into account the characteristics extracted in the encoder part [21,53].

**Figure 7:**
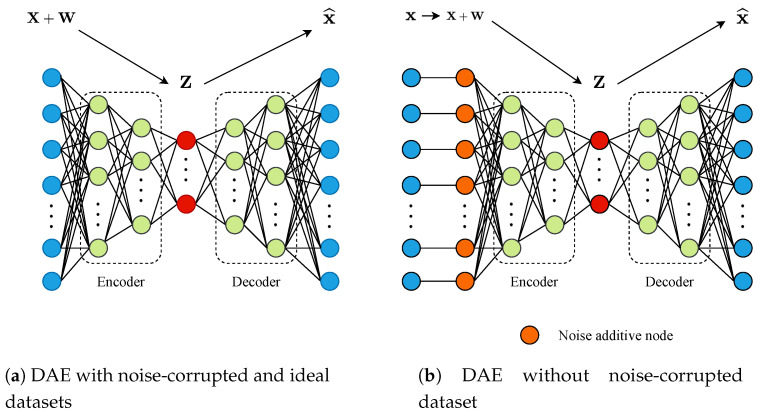
Denoising Autoencoder (DAE) architectures. $x\in R^{m\times 1}$ corresponds to the input data. $w\in R^{m\times 1}$ corresponds to the noise corrupting the input data vector. $z\in R^{k\times 1}$ vector corresponds to the compressed data which is represented in a space with k dimensions. Finally, $x\in R^{m\times 1}$ is the denoised data vector. Nodes in red correspond to the DAE latent space.

A DAE corresponds to an Autoencoder extension which has been conceived to denoise the input signals and recover them without noise. Instead of training the Autoencoder with the same input and output data, it is trained considering noise-corrupted measurements as input data and clean ones as the outputs. In such a context, two different approaches can be performed depending on the available data. If noise-corrupted and clean measurements are available, the DAE architecture can be conceived as a neural network with different layers performing the encoding and decoding processes (see Figure 7a). In the case where noise-corrupted information is not available, the DAE can be still considered if a noise layer is added to its structure, that is, a layer adding noise to the input data. The noise added in this layer corresponds to Additive White Gaussian Noise (AWGN) with zero mean and unit variance (see Figure 7b). It has been adopted due to the fact that BSM1 sensors consider a white zero-mean normally distributed noise [10]. It will show unity variance when normalised by the normalisation layer. It is also worth to notice that the DAE can be implemented considering different types of ANNs such as MLP (the usual structure) or more complex ones like LSTM cells [24,54].

In such a context, the architectures considered in this work are equivalent to the ones adopted in the LSTM-based architectures. They consider a normalisation and denormalisation layers as well as a sliding window with a window length of 4 h. The difference is placed in the neural network where instead of forecasting a concentration value, they will be in charge of denoising the extra input measurements of the LSTM-based IMC and recover a cleaned version of them.

#### 3.2.2. Hyperparameters Tuning

The DAE architectures have been determined performing a grid search where the number of hidden layers or cells, the number of hidden neurons and the learning rate have been set. To perform this search, two datasets have been considered. As it happens with the datasets generated in the LSTM-based architectures hyperparameters tunning process, the datasets considered here have been obtained from the BSM1 model considering a sampling frequency of 1.11 mHz. The noise corrupted dataset corresponds to AWGN noise-corrupted measurements gathered with the sensors implemented in BSM1 model. AWGN noise has been considered since it is the noise introduced by the sensors defined in this model [10]. In the case of the ideal measurements, they are gathered by ideal sensors, that is, the considered measurements are gathered without noise or delays. Both datasets have been generated considering a whole year influent simulation. For that purpose, dry, rainy and stormy influent profiles have been uniformly distributed to generate a dataset corresponding to a whole year of influent measurements. In that sense, once the DAE architectures are obtained they will be considered as the denoising stage of the aforementioned ANN-IMC structures. They will be tested in the real scenario, that is, the BSM1 model considering noise-corrupted measurements.

The obtained architectures correspond to:

- MLP-based DAE: The encoder part is implemented with two hidden layers where the first one has 50 hidden neurons and the second one has 25 hidden neurons. The middle layer of the DAE (the layer of the latent space) has a total amount of 10 hidden neurons. The decoder has the inverse distribution of the encoder, that is, the first layer has 25 hidden neurons while the last one has 50. The learning rate equals to $1\cdot 10^{-3}$.
- LSTM-based DAE: As in the case of the MLP-based DAE, the encoder and decoder parts have two cells. The encoder cells have 50 hidden neurons and 25 hidden neurons respectively. The decoder cells have 25 and 50 hidden neurons. In this case, the learning rate has been set to $5\cdot 10^{-3}$.

Finally, the architectures have been cross-validated adopting the ideal dataset where the 85% of data are adopted to cross-validate the nets and to determine the parameters of the regularisation techniques. The remaining data will be adopted for testing purposes. The regularisation and training techniques adopted in the DAE architectures are the L2 extra penalty ([35], Section 7.1.1) and early stopping. It is also worth to mention that a new regularisation technique can be applied depending on the type of values we are working with. If the DAE is trained with ideal data, that is, noise measurements are not available, a noise layer is added to the DAE structures. This not only helps with the DAE training process, but also with the regularisation: adding noise to the input data corresponds to a data augmentation strategy which is defined as a regularisation technique ([35], Section 7.4). For testing purposes, the noise addition layer is not considered, therefore, the noise-corrupted dataset has to be taken into account. Besides, the noise layer has a multiplicative parameter, the *noise factor* which determines the amount of noise introduced in the noise layer [24]. This new parameter has been also determined in the cross-validation process. Thus, the L2 extra penalty and noise factor have been set to $1\cdot 10^{-4}$ and 0.3 (30% of noise), respectively.

## 4. Results

Results have been computed over the two previously mentioned scenarios: (i) the Ideal Scenario, and (ii) the Real one. In the ideal case, the default PI controlling the $S_{O,5}$ concentration has been substituted by the IMC structure based on ANNs. Specially, two types of ANNs have been considered, the MLPs and the LSTMs. $MLP_{d}$ and $LSTM_{d}$ model the direct relationships between the actuation and controlled variables. $MLP_{i}$ and $LSTM_{i}$ model the inverse relationships of the process under control. The main point of this scenario is placed in the sensors it considers—they sense the different involved concentrations neither adding noise nor delays. This scenario has been previously analysed in Reference [20], however, the IMC was implemented with MLP nets and its stability was not computed.

On the other hand, the real scenario considers the same IMC control strategy as in the ideal case. Nevertheless, the sensors considered here add noise and non-desired behaviours to the different measurements. Since the proposed IMC control strategy is implemented with ANNs, it highly depends on the measured data. For that reason, the controller performance drops when noise corrupted measurements are considered. To overcome this, a new denoising preprocessing stage has been proposed. It considers two different approaches, (i) the PCA and (ii) the DAE. Results will show that the DAE approach is the one offering the best behaviour when compared to the PCA.

Results of each scenario have been computed in terms of the predictions performed by the ANNs, the IMC control performance and its stability. Thereby, these results will show which are the best IMC configurations not only for the ideal scenario, but also for the real ones.

In such a context, the predictions performed by the different ANNs will be compared in terms of the Root Mean Squared Error (RMSE), the Mean Average Percentage Error (MAPE) and the determination coefficient ($R^{2}$):

(4) $$\begin{array}{cc} RMSE & =\sqrt{\frac{1}{N}\sum_{n=1}^{N}{\left(y\left[n\right]-\hat{y}\left[n\right]\right)}^{2}}\\ MAPE & =\frac{1}{N}\sum_{n=1}^{N}\frac{|y\left[n\right]-\hat{y}\left[n\right]|}{y[n]}\\ R^{2} & =1-\frac{\sum_{n=1}^{N}{\left(y\left[n\right]-\hat{y}\left[n\right]\right)}^{2}}{\sum_{n=1}^{N}{\left(y\left[n\right]-\bar{y}\right)}^{2}}, \end{array}$$

where $\bar{y}$ is the mean value of the real values (labels), that is, the ones yielded by the process under control. *N* corresponds to the total number of measurements.

RMSE will show the direct relationship between the predicted values and the real ones provided by the process under control, thus, the lower the metric, the lower the error. However, we are going to deal with different measurements such as the $K_{La,5}$ and the $S_{O,5}$ which are highly heterogeneous. Lets consider the $K_{La,5}$, whose range corresponds to the interval [0,350] day^−1^. The performance of the ANN predicting the aeration coefficient can show high RMSE values whereas its predictions are good enough. This is shown with MAPE. It computes the percentage of error committed in the prediction process. Consequently, this metric will complement the RMSE in the sense that it will tell if errors are large or small. Finally, $R^{2}$ is computed to show if predictions follow the tendency of the different measurements or not: the largest the $R^{2}$, the better the predictions. RMSE and $R^{2}$ metrics have been calculated with normalised data whilst MAPE has been computed with real values to avoid divisions by 0. All the values are computed with the test dataset.

### 4.1. Ideal Scenario Results

#### 4.1.1. Prediction Results

Prediction results of the Ideal Scenario are shown in Table 1, where the RMSE, the MAPE and the $R^{2}$ are computed for the different prediction approaches: the MLP and the LSTM nets. Autoregressive Integrated Moving Average (ARIMA) method [55] has been also considered for comparison purposes. From these three prediction approaches, only the ANNs can be implemented in the IMC control structure: the IMC requires prediction approaches which predict the output values from the input ones (predict $S_{O,5}$ from $K_{L,a}$) and vice-versa [19]. On the other hand, the ARIMA model predicts the output measurements considering the previous observed values [55]. In other words, ARIMA only needs $S_{O,5}$ measurements to predict the ${\hat{S}}_{O,5}$. Even thought, ARIMA results have been shown in Table 1 to get an idea of the ANNs predictions quality. Among the different methods predicting the direct relationship, the one offering the lowest performance corresponds to the $ARIMA_{d}$ method. This was expected since ARIMA generates its model considering only the measurements of a certain variable. Thus, the model generation process becomes a harder task than in the case where more input information is provided (MLP and LSTM nets). For instance, the $MLP_{d}$ net is able to improve the RMSE and MAPE around a 71.91% and 88.47%, respectively. Then, between $MLP_{d}$ and $LSTM_{d}$ nets, the improvement for these metrics equal to a 40% and a 77.42%, respectively. This improvement is motivated by the fact that LSTM networks take into account the dynamic evolution over the time, that is, the time-correlation between measurements. This is achieved due to the memory cell they implement and the processes they perform to the stored information ([35], Section 10.10). In terms of the inverse models, the lowest performance is given by the $MLP_{i}$ method. As observed, the ARIMA method is able to improve the MLP performance, however, it cannot be implemented in the IMC structures by the reasons stated above. Nevertheless, $LSTM_{i}$ overcomes not only the $MLP_{i}$ results, but also the $ARIMA_{i}$ ones. In this case, the improvement achieved by the $LSTM_{i}$ w.r.t. the $MLP_{i}$ corresponds to a 45% in the case of the RMSE, a 25.73% in the case of the MAPE and a 1.01% in the case of the $R^{2}$ metric. As observed, the MAPE improvement considerably differs between the direct and inverse relationships. This is directly related to the complexity of the processes being modelled, the direct process is less complex than the inverse one. Finally, the results obtained here clearly show that LSTM cells are the prediction approach offering the best performance and therefore, they are the ones considered to model the process under control.

**Figure 8:**
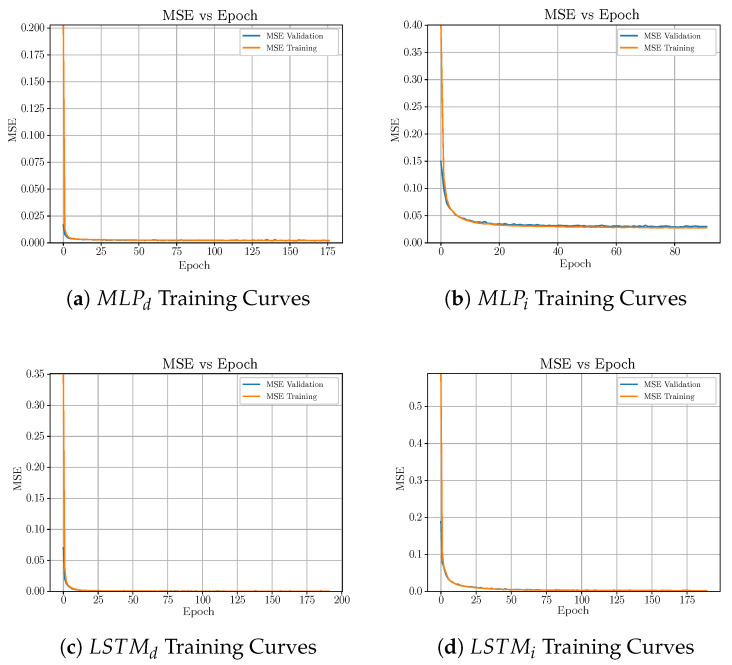
Training curves of the MLP and LSTM structures. Notice that neither of them reach the 500 epochs due to the application of Early Stopping.

Another point which has to be taken into account when dealing with ANNs is the overfitting problem. Although regularisation techniques can be applied to mitigate its effects, one has to analyse the results to determine if overfitting is present or not. In this case, this analysis has been performed by means of the ANNs training curves. These curves show the evolution of the ANNs cost function (Mean Squared Error—(MSE)) along the training process. In this case, two curves are considered: (i) the evolution of the cost function when the subset devoted to training the net is adopted (training curve) and (ii) the evolution when the subset devoted to validate the network is considered (validation curve). When overfitting is committed, there will be a point where the training curve is still decreasing towards zero whereas the validation curve starts to grow up ([35], Chapter 7). This means that the ANN model is memorising the input/output pairs of data instead of generalising. In our case, overfitting is not committed neither in the MLP structures nor in the LSTM ones since there are not any offset between training and validation curves (see Figure 8).

#### 4.1.2. Control Performance

Two tests are computed to determine the control performance of the proposed IMC structure. The former is focused on determining the stability of the IMC structure whereas the latter will determine the control behaviour of the whole structure. The ETFE-based method is considered here to determine which are those frequencies where the proposed IMC structure is robustly stable or not [38,39]. Besides, this stability test will also determine which are the most suitable cut-off frequencies ($\omega_{c}$) of the IMC low-pass filter, which is devoted to filtering non-desired behaviours related to unmodelled dynamics and inversion uncertainties of the process under control [19].

**Figure 9:**
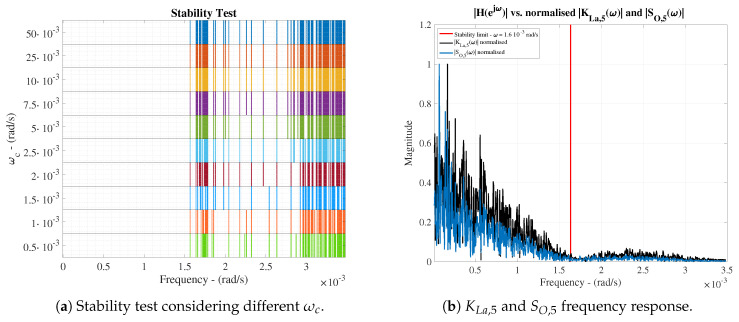
(**a**) shows the stability analysis of the Artificial Neural Network (ANN)-based Internal Model Controller (IMC) for different $\omega_{c}$. Those frequencies showing a color are the ones where the ANN-based IMC fails the stability test. (**b**) shows the frequency response of the controlled variable $S_{O,5}$ and the actuation variable $K_{La,5}$. Notice that the stability limit (vertical red line) corresponds to the one obtained when a cut-off frequency ($\omega_{c}$) equal to $\omega_{c}=10\cdot 10^{-3}\;$ rad/s.

Results of the ETFE-based stability test are shown in Figure 9a where 10 different cut-off frequencies of the IMC low-pass filter are considered. Here, those frequencies where the IMC structure stability cannot be assured are shown. For instance, if the cut-off frequency equals to $\omega_{c}=10\cdot 10^{-3}\;$ rad/s, the IMC structure is marginally stable until $\omega =1.6\cdot 10^{-3}\;$ rad/s. Thus, the stability of the proposed IMC approach can be assured until this frequency due to the fact that the highest frequency component of IMC input signals equals to $\omega =1.5\cdot 10^{-3}\;$ rad/s and it is shown by the $K_{La,5}$ variable (see Figure 9b). When the cut-off frequency is decreased, the stability range is increased, however, the filter actuation will be higher and therefore, the control performance can be degraded.

**Figure 10:**
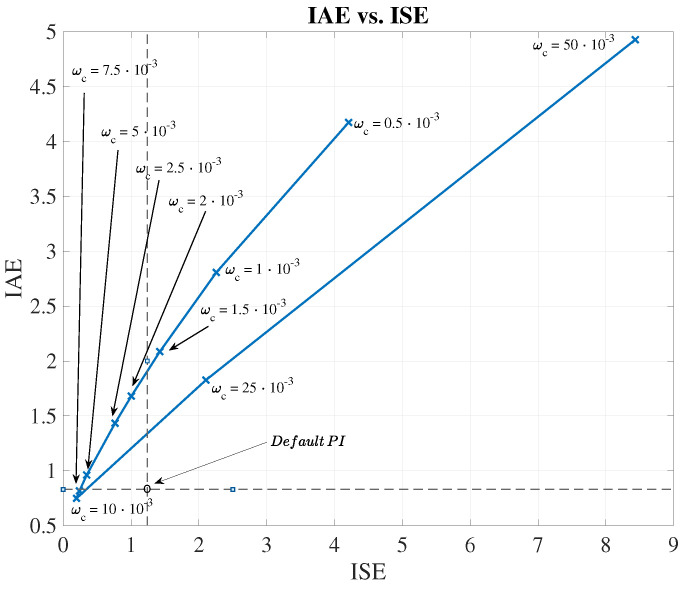
Evolution of Integrated Absolute Error (IAE) and Integrated Squared Error (ISE) as a function of $\omega_{c}$. The different values of IAE and ISE are computed as a function of the $\omega_{c}$. The best $\omega_{c}$ is the one whose IAE and ISE values are close to 0. Notice that this point yields better IAE and ISE values than the default PI controller (∘).

**Figure 11:**
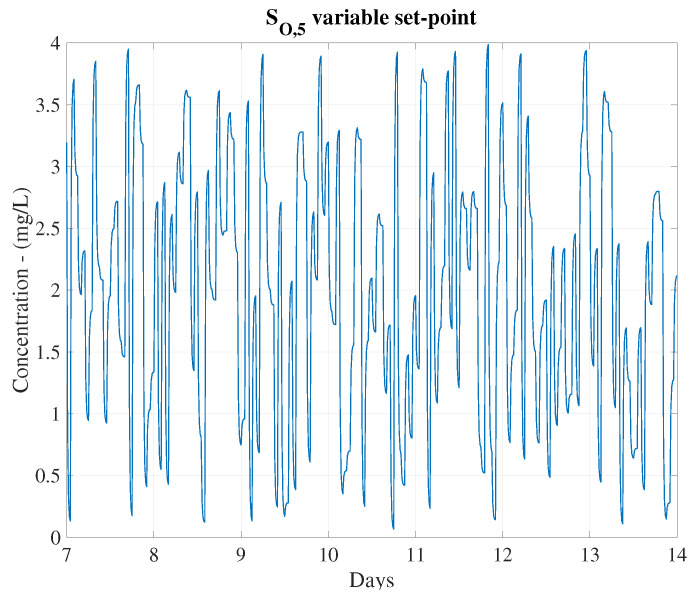
$S_{O,5}$ variable set-point. From now on, this set-point is the one considered to obtain all the results that attain this work. Only from day 7 to 14 are shown since they are the days considered in the performance computation.

This is clearly shown in the control behaviour (see Figure 10), where the evolution of the IAE and the ISE have been computed as a function of the cut-off frequency for a certain variable set-point (see Figure 11). As observed, $\omega_{c}=10\cdot 10^{-3}\;$ rad/s is the optimal cut-off frequency offering the lowest values of the considered metrics: IAE=0.75, ISE=0.20. For instance, if $\omega_{c}$ equals to $2.5\cdot 10^{-3}\;$ rad/s, a worsening in the control performance is produced—IAE and ISE are increased around a 47.76% and 73.44%, respectively.

When comparing the default PI structure with the IMC prediction approach, one can also observe that the proposed LSTM-based IMC structure is able to improve the default PI metrics: IAE=1.24, ISE=0.83. Thus, the highest improvement is again performed when a $\omega_{c}$ of $10\cdot 10^{-3}\;$ rad/s is considered: the IAE and ISE metrics are improved a 39.52% and a 75.90%, respectively.

**Figure 12:**
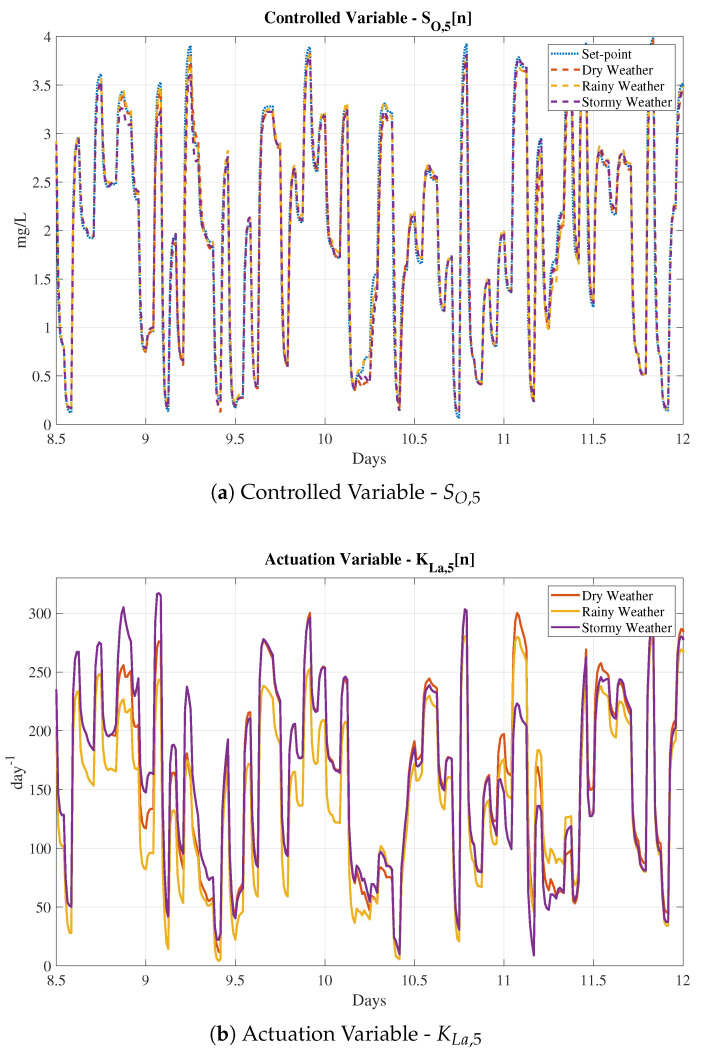
Control behaviour of the LSTM-based IMC controller (controlled and actuation variables are shown in (**a**) and (**b**), respectively). Dry, rainy and stormy weathers have been considered. Notice that only those days where the behaviour of the LSTM-based controller varies are shown.

Finally, the complete structures have been tested adopting the different incoming influents: the dry, the rainy and the stormy ones. Results are shown in Table 2, where it is observed that the proposed control is able to better track the given set-point than the default PI controller does independently of the considered weather. When rain and storm episodes are produced, the amount of influent in the WWTP increases and therefore, the oxygen concentration in the reactor tanks decreases. Moreover, if the variable set-point shows that an increment of this concentration is required, the controller will act consequently adding more oxygen in the fifth reactor tank (see Figure 12). Then, when the LSTM-based IMC is applied, the control is better performed as shown by the IAE and ISE values.

As a summary, this LSTM-based IMC controller has been designed to track a given variational set-point independently of the influent profile entering in the WWTP plant. Results have shown that this IMC controller is able to perform the task it has been designed for better than a PI controller. For instance, the LSTM-based controller is able to improve the PI control behaviour at the expense of increasing the overall costs in some of the cases. However, this performance corresponds to an ideal scenario where neither noise nor undesired behaviours are present. If a real scenario is considered instead, the performance of the LSTM-based IMC controller drops.

### 4.2. Real Scenario Results

In the real scenario, real sensors are used instead of ideal ones. These are characterised by introducing different non-desired behaviours such as delays in the measurements and noise [10]. In such a context, the IMC controller performance will drop if the LSTM nets adopted to model the process under control in the ideal scenario are not modified accordingly to the new circumstances (see Table 3). For instance, their IAE and ISE metrics are now around 1.90 and 0.83, respectively, which represents an average increment in the IAE and ISE around a 63.16% and a 79.52% w.r.t the ideal scenario. Besides, if we compute the difference of the improvements between the ideal and the real scenario, we will observe that the IAE and ISE improvements have been degraded around a 30.49 and 34.83 percentage points, respectively. This change is motivated by the design of the PI and the IMC controller. Although the PI showed a good performance in the ideal scenario, it has not been conceived to deal with real measurements [10]. For that reason, the LSTM-based IMC is able to improve the control behaviour. Moreover, they can be improved even more if actions to mitigate the loss of improvement are considered.

#### 4.2.1. Prediction Performance

The first prediction approach consists in retraining the ANNs to model the process under control considering the effects of noise. In that manner, the ANNs will perform predictions of ideal measurements from noisy ones. Results of this approach are shown in Table 4, where the prediction metrics have been computed once the training process has ended. In addition, the ARIMA performance has been considered again as a baseline although it cannot be implemented in an IMC structure. In this case, the performance of $ARIMA_{d}$, the model in charge of the direct relationship, has been degraded until it shows an RMSE three times bigger than in the ideal case (RMSE=0.374), a MAPE equal to 282.09% and a $R^{2}$ of 0.86. Results of the MAPE clearly show that ARIMA models cannot be considered not only by the aforementioned fact, but also by generating a model not able to deal with noisy signals. It is worth mentioning that $ARIMA_{i}$, the model in charge of forecasting the actuation variable ($K_{La,5}$), is exactly equal to the ideal case. This is motivated by the fact that the actuation variable is not corrupted by noise since it is a value computed by the IMC. Moreover, it actuates directly over the oxygen valve without going through a sensor but through an actuator which adds a delay [10].

**Figure 13:**
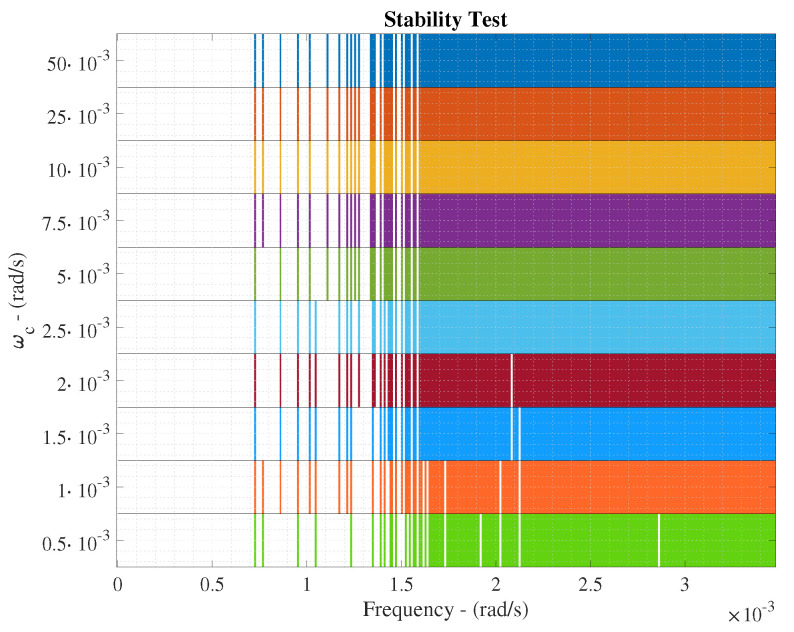
Stability analysis of the LSTM-based prediction structure retrained with noisy data.

MLP and LSTM results show that the prediction performance is degraded w.r.t. the ideal scenario. This effect is expected since the nets have to model the process under control taking into account measurements corrupted by noise. For instance, the RMSE has increased from 0.030 to 0.043 and from 0.045 to 0.101 in the $LSTM_{d}$ and $LSTM_{i}$ nets, respectively. This is translated into a degradation between a 30.23% and a 55.44%. If the performance is only compared in terms of the noise scenario, we will observe that the improvement of LSTMs w.r.t. the MLP equals to a 62.55% and a 58.48% in average for the RMSE and MAPE values, respectively. Analysing the last ones, one will observe that they equal to a 15.85% and a 36.90% for the $LSTM_{i}$ and the $LSTM_{d}$, respectively. This shows that training the neural networks with noisy signals will entail the appearance of high errors and therefore, an incorrect control behaviour. Moreover, these errors can destabilise the whole system as it is shown in Figure 13, where the robust stability of the whole system cannot be assured for frequencies higher than $0.7\cdot 10^{-3}$ rad/s.

In such a context, the denoising stage solutions are proposed with a twofold objective—(i) increase the range where the whole IMC controller is marginally stable and (ii) improve the control behaviour of the whole system. Thus, the performance of this stage will be computed in terms of the denoising accuracy, the stability of the considered denoising approaches and finally the control performance.

The accuracy results are shown in Table 5, where the RMSE, the MAPE and the $R^{2}$ metrics are computed for the PCA-based, the MLP-based and the LSTM-based denoising approaches. As it is observed, the structure offering the best performance is the MLP-SW, which corresponds to the MLP-based DAE implemented with a SW of 4 h. In this case, the accuracy metrics show that this structure is able to offer a RMSE equal to 0.089, a MAPE around a 3.72% and a determination coefficient of 0.99. Besides, there are other approaches showing a good denoising accuracy. For instance, the LSTM-based DAE and the PCA-SW approaches are offering RMSE values equal to 0.091 and 0.131, respectively. In percentage, these values correspond to a worsening of 2.20% and 32.06% w.r.t. the MLP-SW approach. In terms of the MAPE and $R^{2}$, the worsening between the LSTM-SW and the PCA-SW w.r.t the MLP-SW is around 3.2 and 5.6 percentage points for the MAPE and 0.01 units for the $R^{2}$. This shows that among the different structures these three are the ones which can be considered as a denoising tool.

**Figure 14:**
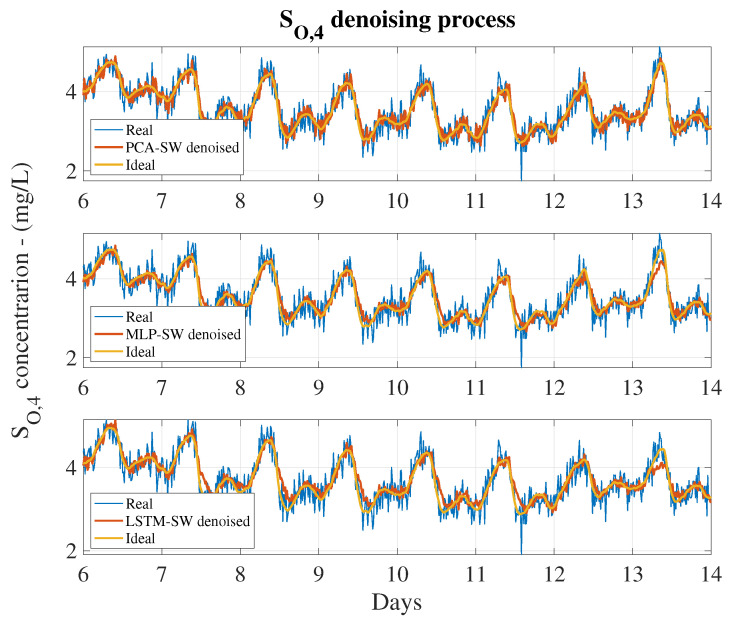
$S_{O,4}$ denoising process. The measured signal is depicted in blue, the denoised and real ones are shown in orange and yellow, respectively.

Let us suppose that a real sensor has measured a $S_{O,4}$ concentration equal to 2.9 mg/L (real measurement + noise). However, the ideal measurement, that is, without noise, corresponds to 2.5 mg/L. To overcome this measurement error, a denoising approach is considered. When applying a simple MLP, the new value will be in the range [2.16–2.83] due to the fact that its MAPE equals to 13.25%. On the other hand, the denoised measurement will be placed in the range [2.41–2.59] when a MLP-SW approach is adopted. Consequently, the lower the MAPE values, the better the denoising approach. For that reason, those approaches showing high MAPE (over 10%) have been directly neglected. The denoising process carried out by the different denosing approaches can be observed in Figure 14, where the noisy, the ideal and denoised $S_{O,4}$ signals are shown for the PCA-SW, MLP-SW and LSTM-SW approaches. Their denoising performance is shown in Table 5.

#### 4.2.2. Control Performance

**Figure 15:**
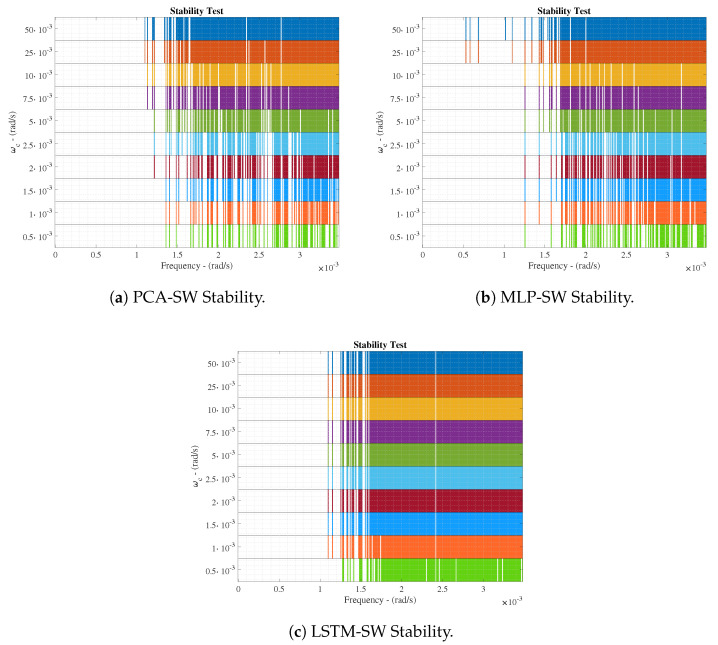
Stability analysis for the different denoising approaches.

Before computing the control performance, the stability of the system has to be analysed. The ETFE-based analysis has shown that none of the different denoising approaches are able to assure a robust stability below the $1.6\cdot 10^{-3}\;$ rad/s, however, they are able to increase the stability margin w.r.t. the LSTM-based nets retrained with noisy signals (see Figure 15). For instance, the retrained LSTM-based nets are able to increase the bandwidth where the controller is able to work from the $0.7\cdot 10^{-3}\;$ rad/s until at least the $1.10\cdot 10^{-3}$ rad/s (when a $\omega_{c}=10\cdot 10^{-3}\;$ rad/s is chosen). Although this frequency is lower than the stability limit of the ideal case, it is worth to notice that the MLP-SW approach is able to offer a greater margin of stability (until the $1.25\cdot 10^{-3}\;$ rad/s) at the same time it only fails the ETFE-based stability test in three frequencies below the $1.6\cdot 10^{-3}\;$ rad/s. Neither the LSTM-SW nor the PCA-SW are able to pass the stability test in more frequencies than the MLP-SW does. For that reason, the MLP-SW approach is the best candidate to be adopted as the principal denoising approach. This will also be confirmed analysing the control behaviour. In such a context, the same $\omega_{c}$ as in the ideal scenario will be considered since the LSTM-based IMC controller is the same.

The control performance of the LSTM-based IMC considering a denoising process has been computed adopting the three most suitable approaches (PCA-SW, MLP-SW and LSTM-SW). The prediction part of this LSTM-based IMC structure corresponds to the LSTM cells considered in the ideal scenario. Therefore, any improvement w.r.t. the results shown in Table 3 will be directly related to the behaviour of the denoising approaches. In that sense, results of the whole system are shown in Table 6. The IAE and ISE metrics show that the control strategy is yielding better results than the default PI. This is directly related to the noise effect: the denoising approaches are filtering the noise of the signals entering in the $LSTM_{d}$ and $LSTM_{i}$ nets. However, the $S_{O,5}\left[n\right]$ and the $S_{O,4}\left[n\right]$ coming from the real plant are noise-corrupted. As a consequence, the mismatch between the real $S_{O,5}\left[n\right]$ and the predicted one will contain noise components. Moreover, DAEs are denoising approaches with losses able to reduce the noise of the signals at expense of recovering them with small variations. Consequently, although denoising techniques have been considered, part of the noise effect is still present in the predictions performed by $LSTM_{d}$ and $LSTM_{i}$. These will entail the generation of $K_{La,5}\left[n\right]$ values which differs from the ones required to track the $S_{O,5}\left[n\right]$ in the best way. Even though, if no denoising stage is considered, the tracking process is worst performed as shown in Table 3.

**Figure 16:**
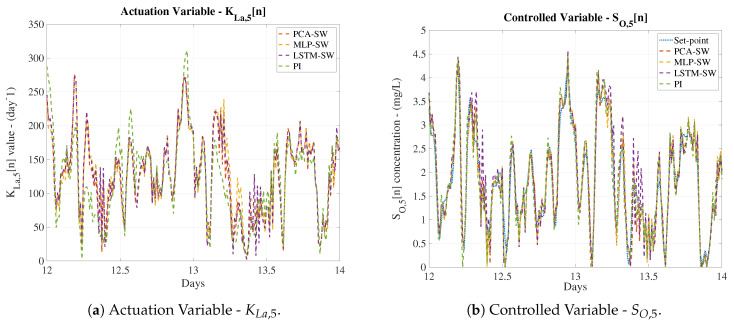
Tracking process of the $S_{O,5}\left[n\right]$ concentration. The three different denoising approaches have been considered, however, the one offering the best tracking is the Multilayer Perceptron-Sliding Window (MLP-SW).

Analysing the numerical results, it is observed that the best improvement w.r.t. the default PI structure is offered by the MLP-SW approach. It achieves an average improvement around a 21.25% for the IAE and a 54.64% for the ISE, while the PCA-SW and the LSTM-SW approaches achieve lower values of the IAE and ISE metrics. These results show that the three considered approaches are valid as a denoising stage previous to the LSTM-based IMC control structure. Not only this, the stability of the control structure has to also be taken into account. As shown in Figure 9a, the MLP-SW is again the denoising approach offering the higher stability margin. Besides, if the controlled signals are compared, we will observe that the MLP-SW approach is the one offering the most accurate signal, that is, the MLP-SW approach is the one performing the best tracking process (see Figure 16). On the other hand, the LSTM-SW approach shows some points where the tracking process differs even more than the PI approach. This fact motivates the adoption of the MLP-SW denoising approach as the main denoising tool and the LSTM-based IMC as the main control structure.

## 5. Conclusions

In this work a new IMC control strategy and a denoising preprocessing stage have been proposed. They have been deployed and implemented over a WWTP where the main objective is to control the dissolved oxygen concentration in the fifth reactor tank. One of the points of this new control approach is placed in the direct and inverse models of the process under control. ANNs have been considered to generate these models instead of generating them as it is done in conventional processes. Since this work is focused on WWTPs, the different measurements (concentrations) can be conceived as time-series showing a high correlation in time. As a consequence, LSTM nets have been proposed in the direct and inverse model generation process due to their ability in modelling highly complex and non-linear processes as well as time-series signals. This approach has been tested over BSM1 framework when ideal sensors are adopted. Results have shown that the proposed LSTM-based IMC is able to improve the default BSM1 PI control strategy metrics, the IAE and ISE, around a 43.28% and a 79.12% in average, respectively. Moreover, an ETFE-based stability test is performed to determine the controller robust stability margin. However, this situation changes when real sensors adding noise and delays to the measurements are adopted. A degradation of the control is performed if no action is proposed to overcome the noise problem.

Two different approaches can be adopted in such a situation, the retraining of the LSTM nets or the adoption of a denoising stage. The former option is simple, it only requires a retraining of the nets adopting noisy measurements instead of ideal ones. In this case, results show that LSTM nets are able to improve the RMSE a 59.81% and a 88.50% when compared with the MLP and the ARIMA approaches modelling the direct relationship between the actuation and control signals. Nevertheless, this entails a degradation of the stability, which cannot be assured for the frequency range of the control input signals. This has been solved considering the denoising stage. Two different approaches have been considered, a PCA solution and the application of DAEs. Among the different DAEs, two different configurations have been considered, the application of MLP and LSTM nets. In addition, both approaches have been tested and implemented considering the adoption of a sliding window of 4 h of WWTP concentrations. In such a context, the denoising accuracy shows that the best denoising approach is offered by the MLP-SW which shows a RMSE, a MAPE and a $R^{2}$ equal to 0.089, 3.72% and 0.99, respectively. Besides, once implemented as a control strategy, results show that the IAE and ISE metrics have been improved w.r.t. the default PI in a 21.25% and a 54.64%, respectively. The robust stability margin has been increased until the point where the whole control structure is robustly stable for nearly all the working frequencies. Only three frequencies in the range between the $1.25\cdot 10^{-3}\;$ rad/s and the $1.6\cdot 10^{-3}\;$ rad/s can compromise the stability.

In this context, the main goal of this new control and denoising approach is not only placed in its performance, but also in its implementation. The LSTM-based IMC as well as the DAE correspond to data-driven approaches which only require pairs of input/output data of the process under control. This entail that they can be applied to control any industrial process as long as data from the process to be controlled are available. Besides, the fact of being based on data motivates the adoption of these approaches as decentralised cloud-based control structures, where the control is performed remotely as a cloud-based service. In addition, another goal of these approaches is that they entail a reduction of the complexity in the control structure design process since the design of denoising filters or the computation of mathematical models are not required.

## Figures and Tables

**Table 1:** Performance of the different prediction approaches. Direct relationship prediction corresponds to the prediction of $S_{O,5}$ whilst inverse relationship corresponds to the forecasting of $K_{La,5}$. Improvement here is only computed in terms of the MLP and LSTM prediction approaches. RMSE is measured in mg/L and days^−1^ in the case of direct and inverse relationships, respectively.

Prediction Performance			
Direct Relationship			
Prediction approach	RMSE	MAPE	$R^{2}$
ARIMA	0.178	32.27	0.97
$MLP_{d}$ [20]	0.050	3.72	0.98
$LSTM_{d}$ [39]	0.030	0.84	0.99
Improvement [%]	40	77.42	1.01
**Inverse Relationship**			
Prediction approach	RMSE	MAPE	$R^{2}$
ARIMA	0.053	5.007	0.97
$MLP_{i}$ [20]	0.082	5.48	0.98
$LSTM_{i}$ [39]	0.045	4.07	0.99
Improvement [%]	45	25.73	1.01

**Table 2:** Control performance–Ideal Scenario. $\omega_{c}=10\cdot 10$${}^{-3}$   rad/s.

PI vs. LSTM-Based IMC Controllers						
	Influent Weather Conditions					
	Dry		Rainy		Stormy	
Structure	IAE	ISE	IAE	ISE	IAE	ISE
PI	1.24	0.83	1.24	0.83	1.24	0.83
$IMC-\omega_{c}$	0.75	0.20	0.66	0.15	0.70	0.17
Improvement [%]	39.52	75.90	46.77	81.93	43.55	79.52

**Table 3:** Control performance—IMC controller of the ideal scenario considering noise-corrupted measurements.

PI vs. LSTM-Based IMC Controllers						
	Influent Weather Conditions					
	Dry		Rainy		Stormy	
Structure	IAE	ISE	IAE	ISE	IAE	ISE
PI	2.02	1.26	1.99	1.24	2.01	1.27
IMC−LSTM	1.91	0.85	1.89	0.82	1.89	0.83
Improvement [%]	5.45	32.54	5.03	33.87	5.97	34.65

**Table 4:** Performance of the different prediction approaches adopting the test dataset. Improvement here is only computed in terms of the MLP and LSTM prediction approaches.

Prediction Performance			
Direct Relationship			
Prediction approach	RMSE	MAPE	$R^{2}$
ARIMA	0.374	282.09	0.86
$MLP_{d}$	0.107	88.34	0.98
$LSTM_{d}$	0.043	36.90	0.99
Improvement [%]	59.81	58.23	1.01
**Inverse Relationship**			
Prediction approach	RMSE	MAPE	$R^{2}$
ARIMA	0.053	5.007	0.97
$MLP_{i}$	0.291	38.40	0.92
$LSTM_{i}$	0.101	15.85	0.99
Improvement [%]	65.29	58.72	7.07

**Table 5:** Performance of the different denoising approaches. The denoising metrics have been computed adopting the average of the denoising process of each input variable. The best denoising approach is in bold.

Denoising Accuracy			
Denoising Approach	RMSE	MAPE	$R^{2}$
PCA	0.171	12.45	0.96
PCA-SW	0.131	9.31	0.98
MLP	0.164	13.25	0.97
**MLP-SW**	**0.089**	**3.72**	**0.99**
LSTM-based DAE	0.091	6.93	0.98

**Table 6:** Control performance–Real Scenario.

PI vs. LSTM-Based IMC Controllers						
	**Influent Weather Conditions**					
	**Dry**		**Rainy**		**Stormy**	
**Structure**	**IAE**	**ISE**	**IAE**	**ISE**	**IAE**	**ISE**
PI	2.02	1.26	1.99	1.24	2.01	1.27
$IMC-DAE_{PCA-SW}$	1.67	0.63	1.68	0.64	1.67	0.64
$IMC-DAE_{MLP-SW}$	1.58	0.57	1.59	0.57	1.57	0.57
$IMC-DAE_{LSTM}$	1.76	0.76	1.77	0.72	1.78	0.74

## Abbreviations

The following abbreviations are used in this manuscript:

ANN	Artificial Neural Network
$ANN_{d}$	Direct model based on ANNs
$ANN_{i}$	Inverse model based on ANNs
ARIMA	Autoregressive Integrated Moving Average
ASM1	Activated Sludge Model N.1
ASM2	Activated Sludge Model N.2
ASM2d	Activated Sludge Model N.2d
AWGN	Additive White Gaussian Noise
$BOD_{5}$	5-day Biological Oxygen Demand
BPTT	Back-propagation Through Time
BSM1	Benchmark Simulation Model No. 1
${\tilde{c}}_{g}$	LSTM Cell-update gate
$c_{n}$	LSTM cell state
$C_{x}$	Covariance matrix
COD	Chemical Oxygen Demand (mg/L)
d[n]	Perturbations entering into the IMC
DAE	Denoising Autoencoder
DL	Denormalisation Layer
DO	Dissolved Oxygen
$e^{\prime }\left[n\right]$	Mismatch between the real and the predicted outputs
ETFE	Empirical Transfer Function Estimation
$f_{g}$	LSTM Forget gate
$f_{s}$	Sampling frequency
$h_{n}$	LSTM output
H(z)	First-order IMC filter
IAE	Integrated Absolute Error
$i_{g}$	LSTM Input gate
IMC	Internal Model Control
ISE	Integrated Squared Error
$K_{La,x}$	Oxygen Transfer Coefficient of the *x*-th reactor tank (day^−1^)
LAL	Linear Activation Layer
LSTM	Long Short-Term Memory Cell
MAPE	Mean average percentage error
ML	Machine Learning
MLP	Multilayer Perceptron
MSE	Mean-squared error
$N_{2}$	Nitrogen gas
$NO_{2}$	Nitrogen Dioxide
NARX	Non-linear Autorregressive Exogeneous model
NL	Normalisation Layer
$O_{g}$	LSTM Output gate
P(z)	Process under control
$P_{dir}\left(z\right)$	Direct model of the process under control
$P_{inv}\left(z\right)$	Inverse model of the process under control
PCA	Principal Component Analysis
PI	Proportional integral controller
*Q*	Flow rate
$R^{2}$	Determination coefficient
r[n]	Reference signal
RNN	Recurrent Neural Network
$S_{NO,x}$	Nitrate-nitrogen concentration in the x-th reactor tank (mg/L)
$S_{NH,x}$	Ammonium concentration in the x-th reactor tank (mg/L)
$S_{NK_{j}}$	Kjeldahl Nitrogen concentration (mg/L)
$S_{O,x}$	Dissolved Oxygen concentration in the x-th reactor tank (mg/L)
SW	Sliding Window
TN	Total Nitrogen (mg/L)
TSS	Total Suspended Solids (mg/L)
u[n]	Actuation signal
$\omega_{c}$	H(z) cut-off frequency
WL	Window length
WWTP	Wastewater Treatment Plant
X	Noise corrupted signals considered in the DAE denoising method
$x_{n}$	LSTM input data
y[n]	Controlled signall
$\hat{y}\left[n\right]$	Output of $P_{dir}\left(z\right)$
$\tilde{y}\left[n\right]$	Input signal of the IMC controller
$Z_{x}$	Mapped noise-corrupted input data
